# TBC1D5 reverses the capability of HIF-2α in tumor progression and lipid metabolism in clear cell renal cell carcinoma by regulating the autophagy

**DOI:** 10.1186/s12967-024-05015-y

**Published:** 2024-02-28

**Authors:** Yu Huang, Zhiyong Xiong, Jianjun Wang, Yafen Gao, Qi Cao, Decai Wang, Jian Shi, Zhixian Chen, Xiong Yang

**Affiliations:** 1 Department of Urology, Union Hospital, Tongji Medical College, Huazhong University of Science and Technology, Wuhan, China; 2 Department of Hepatobiliary Surgery, School of Medicine, Mianyang Central Hospital, University of Electronic Science and Technology of China, Mianyang, China; 3 Department of Anesthesiology, Union Hospital, Tongji Medical College, Huazhong University of Science and Technology, Wuhan, China; 4 Department of Urology, School of Medicine, Mianyang Central Hospital, University of Electronic Science and Technology of China, Mianyang, China; 5 Departments of Pathology, Li Ka Shing Faculty of Medicine, School of Clinical Medicine, The University of Hong Kong, Pok Fu Lam, Hong Kong, China

## Abstract

**Background:**

Clear cell renal cell carcinoma (ccRCC) is known for abnormal lipid metabolism and widespread activation of HIF-2α. Recently, the importance of autophagy in ccRCC has been focused, and it has potential connections with HIF-2α and lipid metabolism. However, the specific regulatory mechanism between HIF-2α, autophagy, and lipid metabolism in ccRCC is still unclear.

**Methods:**

In this study, Bioinformatics Analysis and Sequencing of the whole transcriptome were used to screen our target. The expression of TBC1D5 in renal clear cell carcinoma was confirmed by database analysis, immunohistochemistry, PCR and Western blot. The effects of TBC1D5 on tumor cell growth, migration, invasion and lipid metabolism were examined by CCK8, Transwell and oil red staining, and the mechanism of TBC1D5 on autophagy was investigated by Western blot, fluorescence microscopy and electron microscopy. Chloroquine and rapamycin were used to verified the key role of autophagy in effects of TBC1D5 on tumor cell. The regulatory mechanism of TBC1D5 in renal clear cell carcinoma (RCC) was investigated by shhif-2α, shTBC1D5, mimic, inhibitor, ChIP and Luciferase experiments. The animal model of ccRCC was used to evaluate the biological function of TBC1D5 in vivo.

**Results:**

In this study, TBC1D5 was found to be an important bridge between autophagy and HIF-2α. Specifically, TBC1D5 is significantly underexpressed in ccRCC, serving as a tumor suppressor which inhibits tumor progression and lipid accumulation, and is negatively regulated by HIF-2α. Further research has found that TBC1D5 regulates the autophagy pathway to reverse the biological function of HIF-2α in ccRCC. Mechanism studies have shown that HIF-2α regulates TBC1D5 through hsa-miR-7-5p in ccRCC, thereby affecting tumor progression and lipid metabolism through autophagy.

**Conclusions:**

Our research reveals a completely new pathway, HIF-2α/hsa-miR-7-5p/TBC1D5 pathway affects ccRCC progression and lipid metabolism by regulating autophagy.

**Graphical Abstract:**

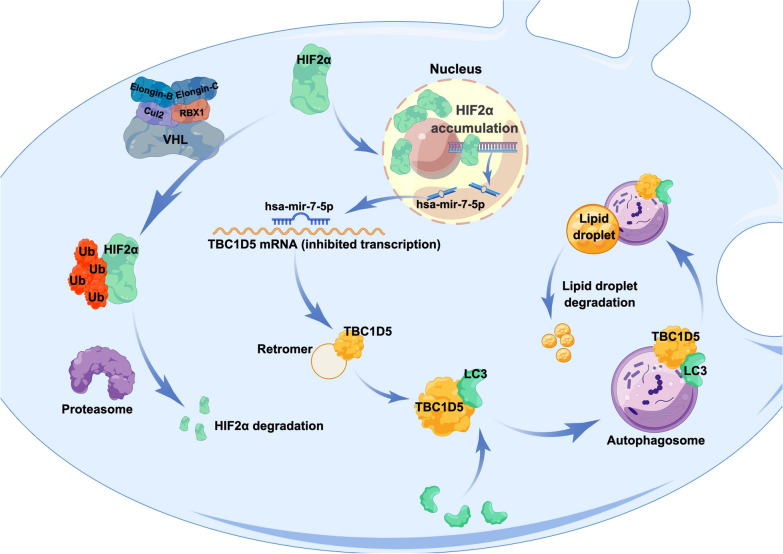

**Supplementary Information:**

The online version contains supplementary material available at 10.1186/s12967-024-05015-y.

## Introduction

Renal Cell Carcinoma (RCC) is one of the most common tumors of the urinary system and ranks in the top 10 new tumor cases, causing > 100,000 deaths annually [1]. Generally, RCC has no apparent symptoms in the early stage. Despite approximately two-thirds of the patients with newly diagnosed RCC being in the relatively early stage that can be managed using timely surgical treatment, the remaining one-third are initially diagnosed with lymph node or distant metastasis, and the prognosis deteriorates rapidly [2, 3]. Clear cell renal cell carcinoma (ccRCC), the most common histologic RCC classification (70–75%), is characterized by a massive accumulation of lipid droplets within tumor cells [4, 5].

The most significant ccRCC molecular change is the inactivation of the tumor suppressor gene von Hippel Lindau (VHL) located on chromosome 3p [6]. Previous studies have shown that approximately 90% of ccRCC lesions contain VHL gene changes, including somatic mutations, methylation changes, and genetic deletions [7]. As an E3 ubiquitin ligase, inactivating the VHL gene increased the stability of α-subunits of oxygen-dependent hypoxia-inducible factor (HIF), promoted the constitutive expression of HIF-1α and HIF-2α, and further induced the activation of hypoxia-related genes, such as VEGF, thus promoting tumor progression [8, 9]. Notably, both HIF-1α and HIF-2α act as transcription factors, and although they have common target genes, they display distinct functions in ccRCC. HIF-1α expression is frequently absent during tumor progression, and sequencing data are consistent with clinical data, suggesting that HIF-1α helps inhibit tumor growth. Conversely, HIF-2α is consistently expressed in VHL-inactivated ccRCC. HIF-2α is crucial for the growth of ccRCC xenografts in vitro, and the metabolic changes it mediates directly promote tumor progression [10, 11].

ccRCC’s characteristic morphological change is the increased accumulation of intracellular lipid droplets in tumor cells [12]. These abnormal lipid droplets can improve the stability of the endoplasmic reticulum and reduce its stress response to cytotoxicity so that tumor cells can maintain good viability under the stimulation of nutrition restriction, hypoxia, drugs, and other ER homeostasis disturbances, promoting tumor progression [11]. The abnormal lipid droplets are closely regulated by HIF-2α and its associated pathway [13]. However, the specific mechanisms of HIF-2α dependent lipid storage remain unclear.

Autophagy is a ubiquitous and highly conserved major catabolic process in eukaryotes [14, 15]. It degrades and recycles proteins and organelles and is highly involved in intracellular lipids regulation [16–18]. Autophagosomes can encapsulate and degrade lipid droplets, releasing free fatty acids into the cytoplasm and generating ATP for energy supply [19]. Inhibiting autophagy increased triglyceride and lipid droplets in vitro and in vivo. When autophagy-related genes were knocked out to induce loss of autophagy, significantly decreased triglyceride catabolism was observed. Lipid synthesis and storage are increased in tumor cells; therefore, autophagy affects tumor progression by regulating lipid metabolism [20, 21]. In ccRCC, the level of autophagy is relatively low in tumors compared with adjacent normal tissues, and low autophagy often indicates a worse prognosis [22–24]. Notably, both HIF-2α and autophagy pathways are highly connected to ccRCC’s lipid metabolism, and few studies have explored the potential linkage between HIF-2α and autophagy. However, none has explored the specific mechanism in ccRCC [25].

TBC1D5, a TBC family protein, is a critical retromer component, mediating the reverse transport of target molecules from endosomes to the Golgi apparatus [26]. TBC1D5 is closely involved in autophagy in several ways. The TBC1D5 molecule contains two LC3-acting elements (LC3-interacting region, Lir), and both are involved in directly binding TBC1D5 and MAP1LC3, further participating in forming autophagosomes from the initial stage [27]. TBC1D5 can also regulate the initiation of mitophagy [28]. However, very few studies have focused on TBC1D5’s roles in tumors, and it has never been reported in ccRCC.

In summary, the effect of HIF-2α in promoting lipid accumulation in ccRCC has been reported. However, the specific mechanism through which HIF-2α regulates lipid metabolism by regulating autophagy and further influences tumor progression in ccRCC has not been reported. We focused on this problem and selected the autophagy-related gene TBC1D5 as the entry point, revealing for the first time that HIF-2α could affect lipid metabolism by inhibiting autophagy in ccRCC. This provides a new understanding of ccRCC’s abnormal lipid metabolism and therapy.

## Results

### TBC1D5 as biomarkers in ccRCC connecting HIF-2α and autophagy

**Fig. 1:**
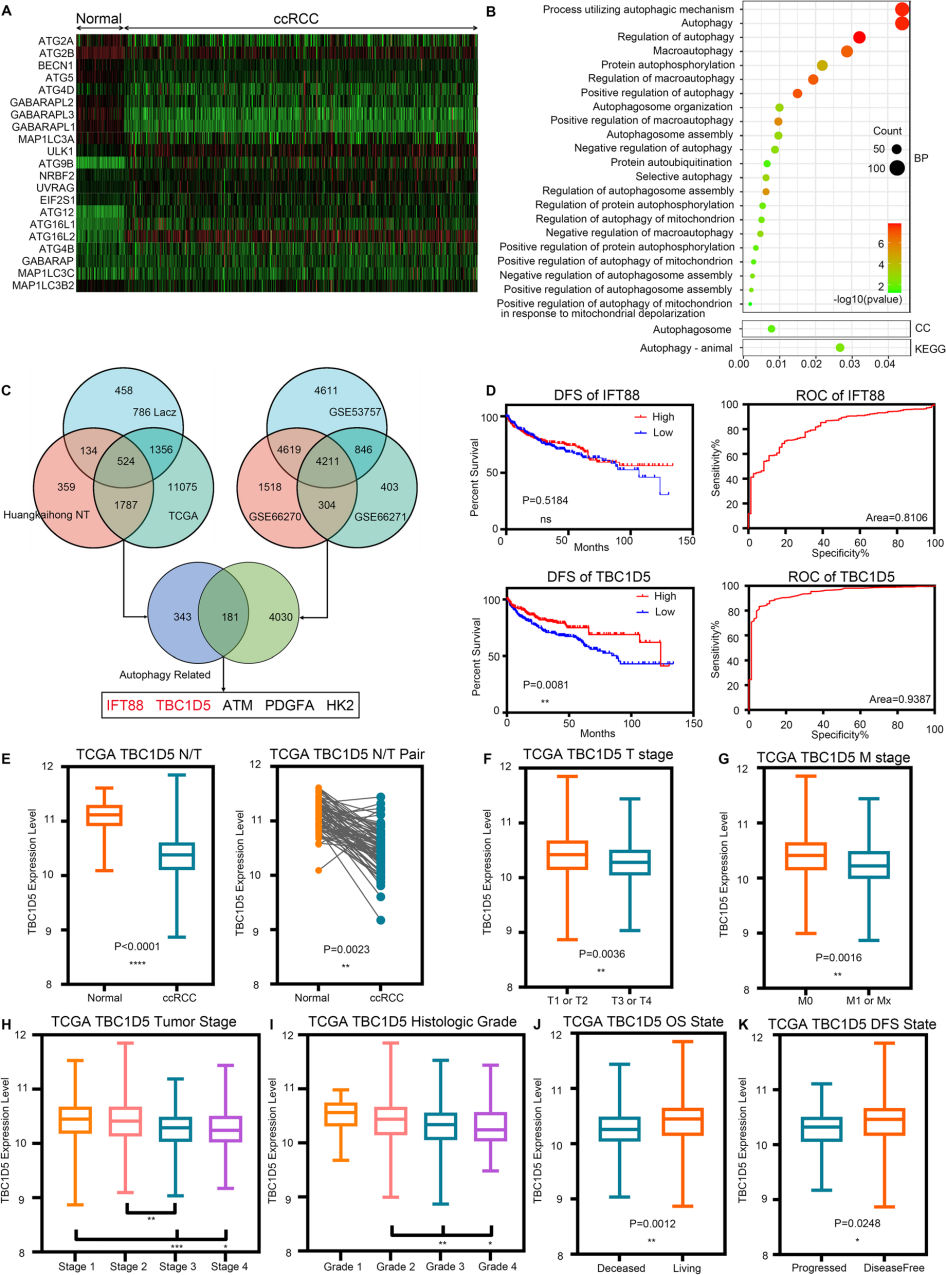
TBC1D5 acted as diagnostic and prognostic biomarkers in ccRCC connecting HIF-2α and autophagy. **A** Heatmap of the expression profiles of 21 autophagy-related different expressed genes (DEGs) in the TCGA-KIRC. **B** Gene Ontology (GO) and Kyoto Encyclopedia of Genes and Genomes (KEGG) pathway analyses of DEGs that significantly correlated with HIF-2α indicated these DEGs enriched in multiple autophagy-related pathways. **C** Venn diagram for DEGs showing autophagy and HIF-2α related genes based on TCGA-KIRC, sequencing data of HIF-2α knockdown RCC cell line, sequencing data of ccRCC tissue, and three independent Gene Expression Omnibus (GEO) datasets. **D** Kaplan–Meier curves of disease-free survival (DFS) and ROC curves for two selected genes, TBC1D5 and AFT88. **E** The mRNA levels of TBC1D5 in paired or unpaired tumor tissue and paracancer tissue. **F**–**H** Patients with relatively high-risk T, M, and tumor stages have lower expression of TBC1D5. **I** The patients with Clear cell renal cell carcinoma (ccRCC) of higher histologic grade also experience a lower expression of TBC1D5. **J**, **K** Deceased patients and progressed patients have a lower expression of TBC1D5. *P < 0.05, **P < 0.01, ***P < 0.001, ****P < 0.0001

Since HIF-2α and autophagy pathways are critical in RCC, we explored their potential in RCC. First, we explored the expression difference of all autophagy-related genes listed on the Kyoto Encyclopedia of Genes and Genomes (KEGG) pathway map (map04140) in the Cancer Genome Atlas (TCGA)-Kidney Renal Clear Cell Carcinoma (TCGA-KIRC). Among all 36 listed genes, the expression level of 21 genes significantly differed between ccRCC and normal adjacent tissue, with nine and 12 genes down-regulated in the normal adjacent tissue and ccRCC, respectively (Fig. 1A). These differentially expressed genes (DEGs) indicated the potential alteration of the autophagy pathway during ccRCC’s development and progression.

We performed Gene Ontology (GO) and KEGG analyses using DEGs of TCGA-KIRC that significantly correlated with HIF-2α, to further explore if a HIF-2α change in ccRCC is associated with autophagy’s alteration. HIF-2α correlated DEGs were enriched in 24 different autophagy-related GO and KEGG terms, involving multiple aspects of autophagy (Fig. 1B). We also selected two ccRCC cell lines, 786-O, and A498, to experimentally verify that silencing HIF-2α increased autophagy levels (Additional file 1: Fig. S1).

To elucidate the specific interaction of HIF-2α and autophagy, sequencing HIF-2α knockdown RCC cell line 786-O and ccRCC tissue data, TCGA-KIRC, three independent ccRCC Gene Expression Omnibus (GEO) gene sets, and the five remaining autophagy-related genes based on their GO database annotations were used to screen the potential target (Fig. 1C). Next, we used Kaplan–Meier curves based on the TCGA-KIRC database for further selection. Two genes significantly differed in overall survival (OS) based on the different expression levels (Additional file 2: Fig. S2). Notably, only TBC1D5 can affect the ccRCC’s disease progression-free survival (DFS) with better area under the curve of the Receiver Operator Characteristic (ROC) curve, indicating TBC1D5 as a potential diagnostic ccRCC indicator (Fig. 1D). Consequently, TBC1D5 was chosen as the target molecule for in-depth study.

Next, bioinformatics was used to analyze TBC1D5’s expression level and TCGA-KIRC’s clinical parameters. TBC1D5’s expression level was lower in ccRCC than in normal tissues, regardless of N/T paired comparisons (Fig. 1E). Coincidentally, survival curve analysis showed that high TBC1D5 expressions predicted better OS (P = 0.0009) and DFS (P = 0.0081) (Fig. 1F). TCGA-KIRC samples were divided into different subgroups based on various clinical parameters. TBC1D5 expression was lower in tumor specimens with more advanced T stage (T3 or T4), M stage (M1), tumor stage, and histological grade. In addition, TBC1D5 expression was lower in deceased or progressed patients compared with living or disease-free patients. Moreover, TBC1D5 expression is significantly associated with multiple clinicopathological parameters, including age, TNM stage, and histological grade (Table 1). TBC1D5 levels can also affect the OS and DFS in different subgroups of ccRCC’s TNM stage, tumor stage, and histologic grade (Additional file 3: Fig. S3). Further, TBC1D5 expression level in normal adjacent tissue of different malignant degree is comparable (Additional file 4: Fig. S4).

**Table 1:** Association between TBC1D5 mRNA expression and clinicopathological parameters of patients with ccRCC

Patient characteristics				
Parameter	TBC1D5 mRNA expression			P-value
	No	Low (n = 265)	High (n = 266)	
Age				
≤ 60	263	113	150	
> 60	268	152	116	0.002
Tumor stage				
Stage 1 or 2	322	139	183	
Stage 3 or 4	209	128	81	0.000
T stage				
T1 or T2	340	150	190	
T3 or T4	191	115	76	0.000
N stage				
N0	239	116	123	
N1 or NX	292	149	143	0.505
M stage				
M0	441	204	237	
M1 or MX	90	60	30	0.000
Histologic grade				
< 8	241	103	138	
≥ 8	290	164	126	0.002
Gender				
Acinar type	187	105	82	
Other subtype	344	160	184	0.034
Primary tumor side				
No	254	136	118	
Yes	277	129	148	0.108

### Relatively low expression of TBC1D5 in ccRCC

**Fig. 2:**
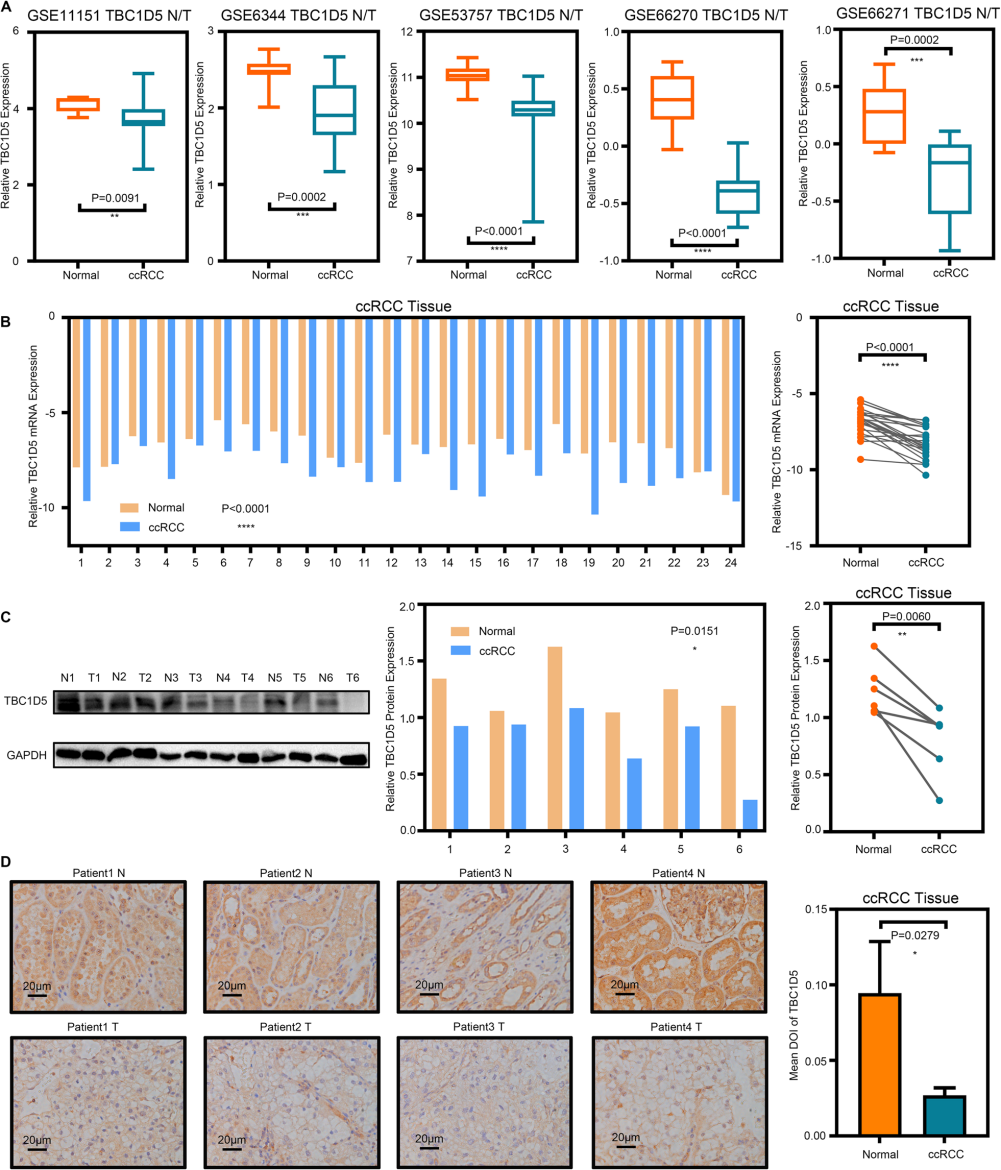
Relatively low expression of TBC1D5 in ccRCC. **A** Verification of the TBC1D5 gene signature in five external cohorts from GEO datasets, the mRNA levels of TBC1D5 were compared between tumor tissue and paired paracancer tissue. **B** The mRNA levels of TBC1D5 in 24 pairs of clinical specimens pathologically diagnosed as ccRCC and paracancer tissues. **C** TBC1D5 expression was evaluated in six pairs of pathologically diagnosed as ccRCC and paracancer tissues by using Western Blot. **D** Immunohistochemistry staining of TBC1D5 was performed in the tumor sections of ccRCC and normal tissues and further exhibited by semi-quantification. Scale bar: 20 μm. *P < 0.05, **P < 0.01, ***P < 0.001, ****P < 0.0001. *IOD* integral optical density

We further confirmed TBC1D5’s expression level in ccRCC. In Imprimis, following TCGA-KIRC, TBC1D5 had relatively low expressions in ccRCC compared with normal kidney tissue in five independent GEO datasets (Fig. 2A). In 24 clinical specimens pathologically diagnosed as ccRCC and their corresponding normal adjacent tissue, TBC1D5’s mRNA level was lower in tumor samples (Fig. 2B). The protein level of TBC1D5 expression was similar to that of mRNA. Western blot using proteins extracted from six ccRCCs and their corresponding normal adjacent tissue showed that ccRCC tissue expressed a lower TBC1D5 protein level (Fig. 2C). This result was further confirmed through immunohistochemical staining of TBC1D5 on four tumor-normal paired slices (Fig. 2D). Overall, data from TCGA-KIRC, the GEO dataset, and results from our multiple experiments confirmed that the mRNA and protein expression of TBC1D5 is relatively low in ccRCC.

### TBC1D5 acted as a tumor suppressor, suppressed ccRCC progression, and reduced lipid accumulation in ccRCC

**Fig. 3:**
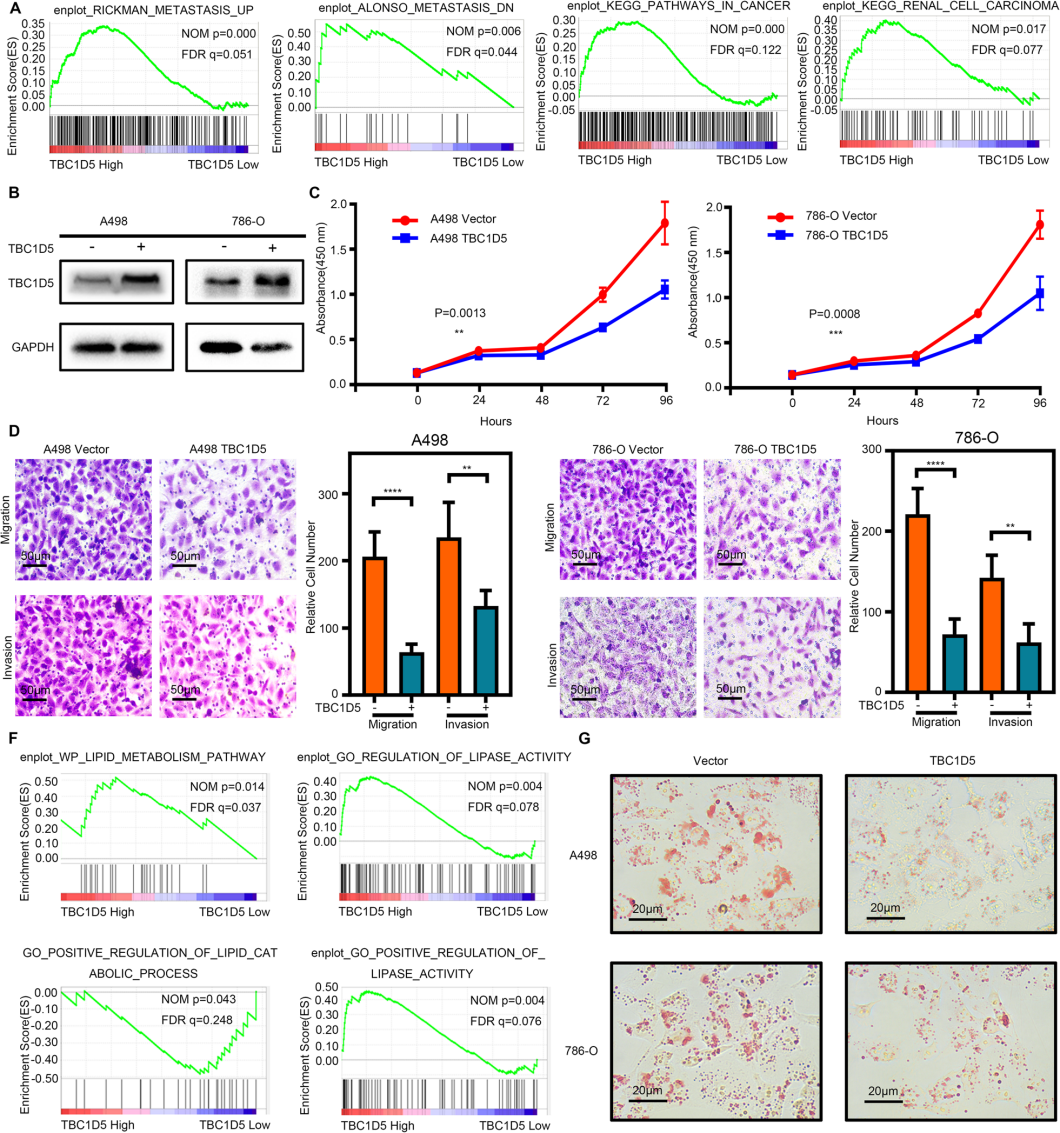
TBC1D5 acted as a tumor suppressor, suppressed ccRCC progression, and reduced lipid accumulation in ccRCC. **A** Gene Set Enrichment Analysis (GSEA) of high and low TBC1D5 expression groups in the Cancer Genome Atlas (TCGA) database correlated with metastasis and renal cell carcinoma. **B** Western blot analysis was used to verify TBC1D5 overexpression at the protein level. **C** The effect of TBC1D5 overexpression on A498 and 786-O cell proliferation was measured using CCK-8 assay. Five microscopic fields were chosen at random and averaged. **D**, **E** The effect of TBC1D5 overexpression on invasion and migration was analyzed using Transwell assays in A498 and 786-O cells, respectively. The histogram represents the relative cell number. Five microscopic fields were selected at random and averaged. Scale bar: 50 μm. **F** GSEA of high and low TBC1D5 expression groups in the TCGA database correlated with lipid metabolism. **G** TBC1D5 overexpression significantly decreased lipid droplet accumulation in ccRCC cells tested with oil red staining. Scale bar: 20 μm. *P < 0.05, **P < 0.01, ***P < 0.001, ****P < 0.0001

Due to the significantly different expression levels in ccRCC and normal kidney tissue, TBC1D5 is likely to affect ccRCC’s biological function. GSEA analysis using the TCGA-KIRC database indicated that TBC1D5 was highly involved in tumor metastasis, cancer, and renal cell carcinoma-related pathways in ccRCC (Fig. 3A). This result revealed the close relationship between TBC1D5 and ccRCC development. To confirm this, we used lentivirus to construct two ccRCC cell lines 786-O and A498 with stable TBC1D5 overexpression since TBC1D5 has a relatively low expression in ccRCC (Fig. 3B). Following Cell Counting Kit-8 (CCK8) experiments, TBC1D5 overexpression significantly reduced the proliferation ability in the 786-O and A498 cell lines (Fig. 3C). The transwell experiment showed a similar tendency, such that cells with TBC1D5 overexpression showed weaker migration and invasion ability (Fig. 3D, E).

As the characteristic morphological change of ccRCC, intracellular lipid droplet accumulation is critical for ccRCC’s normal biological function. Previous GSEA analysis also indicated the function of TBC1D5 in lipid metabolism and the positive regulation of lipid catabolic process and lipase activity in ccRCC (Fig. 3F). Therefore, TBC1D5 expression affects the lipid metabolism in ccRCC. Oil red staining results verify this; lipid droplet accumulation in ccRCC cells was significantly decreased by TBC1D5 overexpression (Fig. 3G and Additional file 5: Fig. S5A). Overall, TBC1D5 suppressed the proliferation, migration, invasion, and lipid droplet accumulation of ccRCC, suggesting TBC1D5 is a potential tumor suppressor.

### TBC1D5 regulates tumor progression and lipid accumulation by influencing autophagy in ccRCC

Autophagy can deeply affect ccRCC progression in various ways, and autophagic degradation of lipid droplets significantly suppresses the progression of ccRCC. In the previous screening process, we selected autophagy-related genes based on the annotations from the GO database and TBC1D5, including autophagy, macroautophagy, and autophagosome, suggesting the close relationship of TBC1D5 and autophagy. Consequently, TBC1D5 overexpression can also regulate lipid metabolism in ccRCC.

**Fig. 4:**
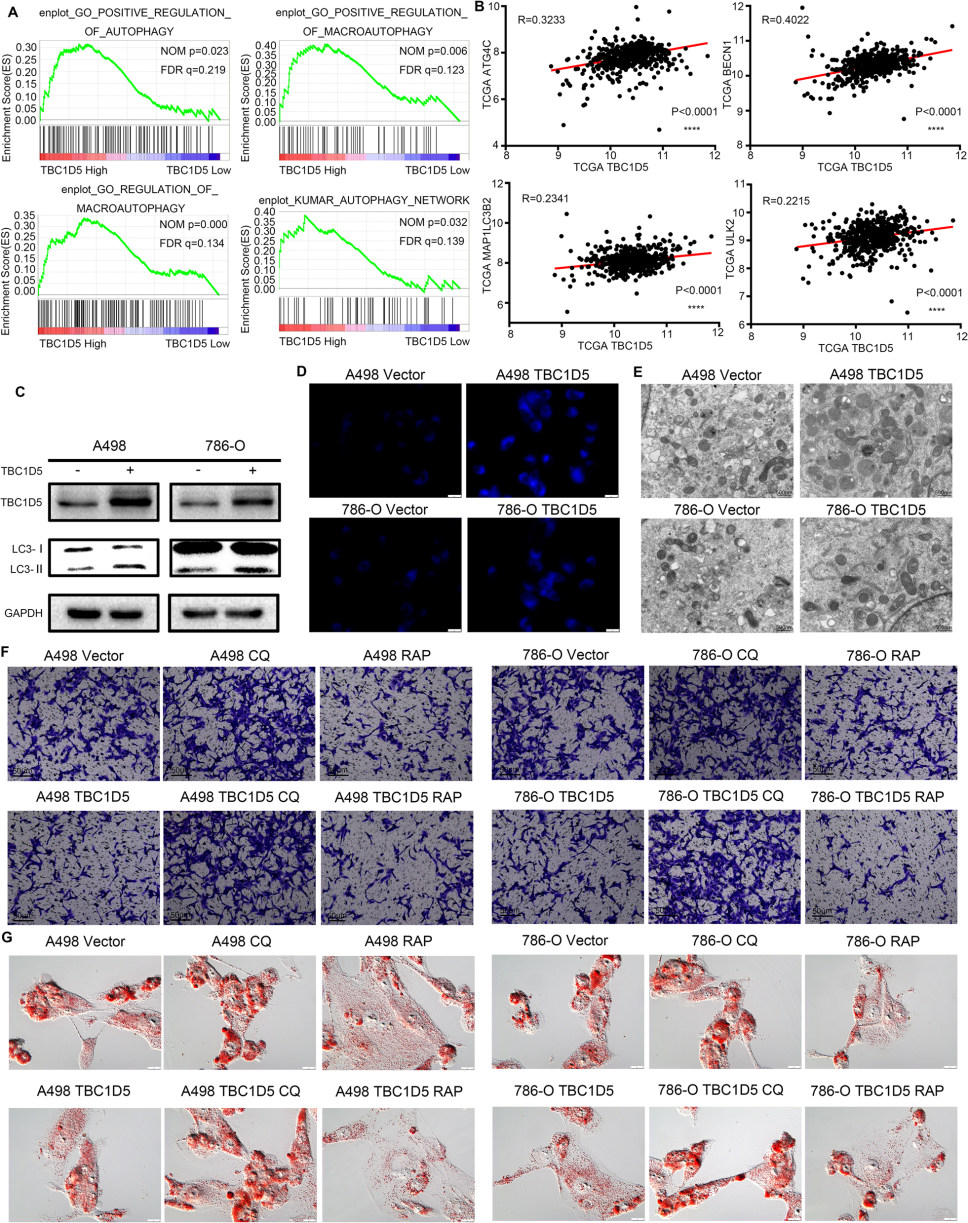
TBC1D5 regulates lipid accumulation by influencing autophagy in renal cancer cells. **A** GSEA of high and low TBC1D5 expression groups in the TCGA database correlated with multiple autophagy-related pathways. **B** Correlation between the mRNA expression level of autophagy-related genes (ATG5, BECN1, LC3, and ULK2) and TBC1D5. **C** After overexpression of TBC1D5, the protein level of LC3 was detected using Western Blot. **D**, **E** Autophagy levels were detected using fluorescence and electron microscopy after TBC1D5 overexpression. Scale bar: 100 μm and 500 nm. **F** The migration ability of A498 and 786-O cells after promoting and inhibiting autophagy treatments was detected using Transwell. Scale bar: 50 μm. **G** The lipid content of A498 and 786-O cells after promoting and inhibiting autophagy treatment as well as TBC1D5 overexpression was detected using an oil red assay. Scale bar: 100 μm. ****P < 0.0001. *CQ* chloroquine, *RAP* rapamycin, *OE* overexpression

Gene Set Enrichment Analysis (GSEA) based on the TCGA-KIRC dataset was conducted to further explore this relationship in ccRCC. GSEA demonstrated that TBC1D5 was involved in the positive regulation of autophagy, macroautophagy, and the autophagy network (Fig. 4A). Moreover, the TBC1D5 expression level positively correlates with several autophagy-related markers, such as ATG4C, BECN1, MAP1LC3B2, and ULK2 in ccRCC (Fig. 4B). In stable TBC1D5 overexpression of ccRCC cell lines 786-O and A498, the protein expression of LC3-II was significantly elevated, demonstrating the promotion function of TBC1D5 on autophagy in ccRCC (Fig. 4C). Consequently, the autophagy assay kit and transmission electron microscopy showed similar results as the stronger fluorescence signal and more autophagosomes observed on TBC1D5 overexpression cells (Fig. 4D, E). Next, autophagy blocker chloroquine and autophagy promoter rapamycin were used to explore further the importance of autophagy in the suppression effect of TBC1D5 in ccRCC (Fig. 4F). Compared with the control group, chloroquine can promote cell migration and lipid accumulation in ccRCC, whereas rapamycin gives the opposite effect. In TBC1D5 overexpressed ccRCC cells, chloroquine could reverse the inhibitory effect of cell migration and reduce lipid droplets caused by TBC1D5 overexpression. However, in synergy with TBC1D5 overexpression, rapamycin inhibits ccRCC migration and lipid droplet reduction (Fig. 4G and Additional file 5: Fig. S5B). These results showed that TBC1D5 can regulate the level of autophagy in ccRCC, and autophagy is critical for TBC1D5’s inhibitory effect in ccRCC.

### TBC1D5 reverses the tumor-promoting action and lipid accumulation of HIF-2α in ccRCC

**Fig. 5:**
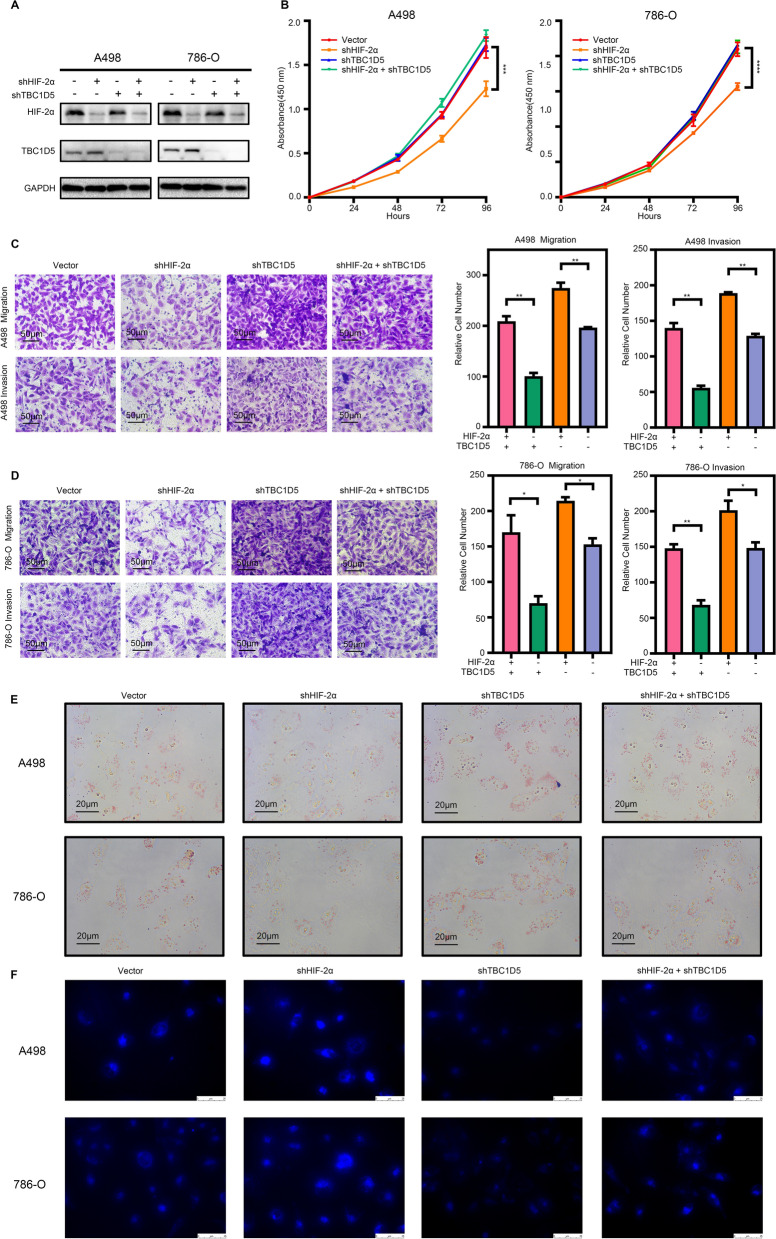
TBC1D5 reverses the tumor-promoting action and metabolic reprogramming of HIF-2α in ccRCC. **A** HIF-2α and TBC1D5 protein levels in transfected cell lines are shown by western blotting. **B** Cell proliferation curves based on CCK8 assays are shown for TBC1D5 knockdown and/or HIF-2α knockdown on A498 and 786-O cell lines. Five microscopic fields were selected at random and averaged. **C**, **D** The effect of TBC1D5 knockdown and/or HIF-2α knockdown on invasion and migration was analyzed using Transwell assays in A498 and 786-O cells, respectively. Scale bar: 50 μm. **E** The effect of TBC1D5 knockdown and/or HIF-2α knockdown on lipid metabolism was analyzed using oil red staining. Scale bar: 20 μm. **F** The effect of TBC1D5 knockdown and/or HIF-2α knockdown on autophagy was analyzed using fluorescent staining. Scale bar: 50 μm. *P < 0.05, **P < 0.01, ***P < 0.001, ****P < 0.0001

As the potential critical gene that links the HIF-2α and TBC1D5 pathway, functional rescue experiments were conducted to illustrate further the impact of TBC1D5 in the HIF-2α mediated tumor progression and lipid accumulation in ccRCC. Since HIF-2α negative regulates TBC1D5expression, we constructed RCC cell lines 786-O and A498 with stable TBC1D5 and HIF-2α knockdowns using shRNA (Fig. 5A). CCK8 experiments showed that HIF-2α knockdown significantly inhibits cell proliferation ability (Fig. 5B). TBC1D5 knockdown alone showed no significant change; however, TBC1D5 and HIF-2α knockdowns simultaneously significantly abolished the inhibitory effect of ccRCC proliferation caused by only HIF-2α knockdown. The migration and invasion ability measured using transwell assay showed the same tendency (Fig. 5C, D). The migration and invasion ability of A498 and 786-O cells was significantly reduced after HIF-2α knockdown; however, further TBC1D5 knockdown on this basis partially reversed this effect (Figs. 5C, D). Through these experiments, we proved that TBC1D5 is a gene regulated by HIF-2α in ccRCC, inhibiting HIF-2α promoted proliferation, migration, and invasion ability of tumor cells and is vital in suppressing cancer.

Furthermore, we performed oil red staining on the previously mentioned cell lines. The knockdown of TBC1D5 significantly increased lipid accumulation; the lipid accumulation in cells was significantly reduced after HIF-2α knockdown, and the knockdown of both HIF-2α and TBC1D5 can reverse the decreased lipid accumulation caused by the knockdown of HIF-2α alone (Fig. 5E and Additional file 5: Fig. S5C). Notably, the above results demonstrated that in ccRCC, TBC1D5 is crucial in the lipid metabolism of tumor cells under the regulation of HIF-2α. Finally, the autophagy level was analyzed using fluorescent staining on the previously mentioned cell lines. The autophagy level was significantly increased after HIF-2α knockdown, TBC1D5 knockdown significantly decreased the autophagy level, and the knockdown of both HIF-2α and TBC1D5 reversed the increased autophagy caused by knockdown of HIF-2α alone (Fig. 5F). The results show that TBC1D5 is crucial in regulating ccRCC lipid metabolism and tumor progression mediated by HIF-2α. We can also speculate that this process is mainly regulated by HIF-2α related autophagy reversed with TBC1D5.

### HIF-2α inhibits the expression of TBC1D5 in ccRCC through hsa-mir-7-5p

**Fig. 6:**
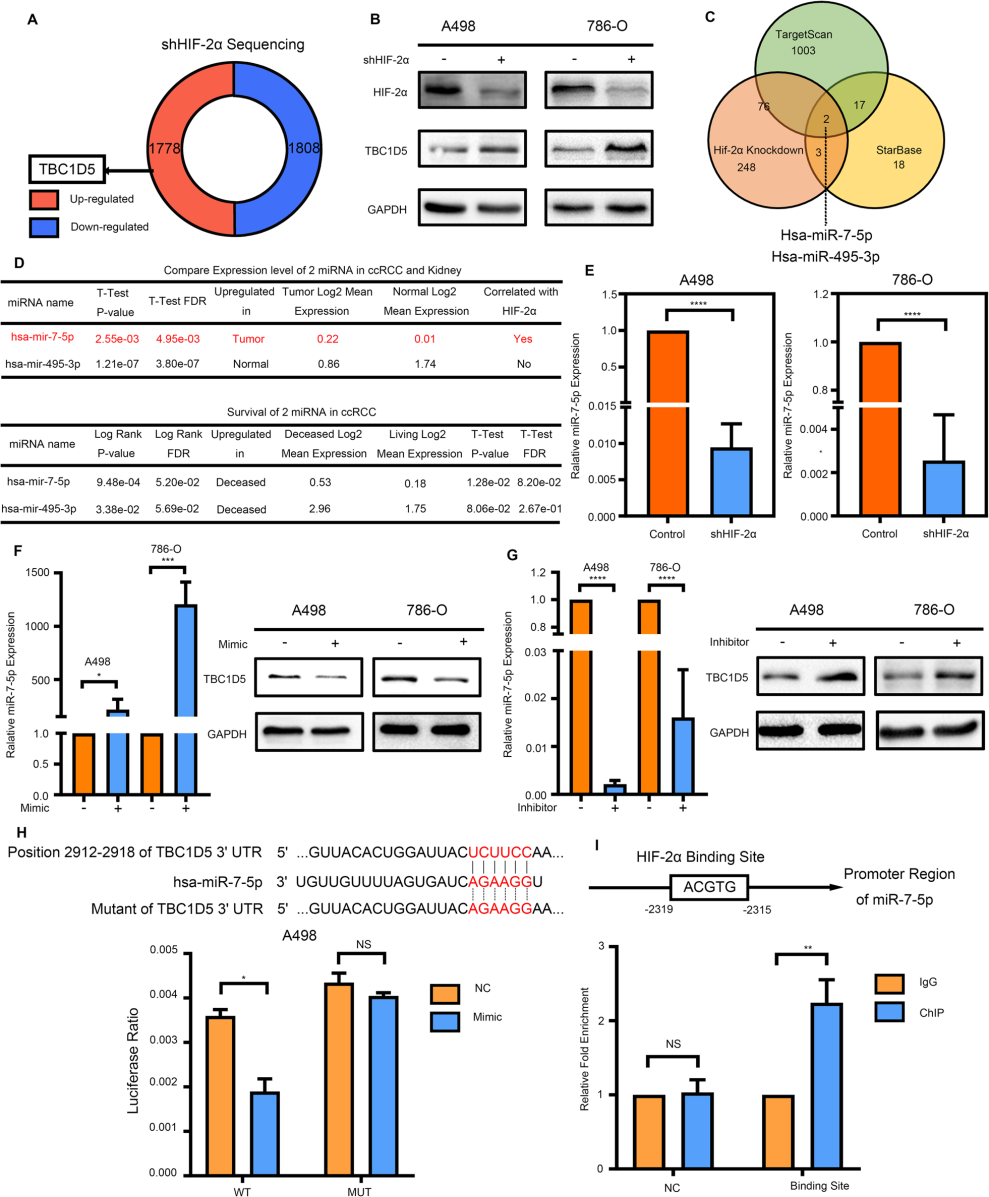
HIF-2α inhibits the expression of TBC1D5 in ccRCC through hsa-mir-7-5p. **A** The expression of TBC1D5 was significantly elevated in our screening datasets containing the sequencing data of HIF-2α knockdown RCC cell line 786-O. **B** After the HIF-2α was knocked down, the TBC1D5 protein level was detected using Western Blot. **C** The sequencing data, TargetScan, and Starbase were used to screen the potential miRNA. **D** Further screening of two selected miRNA based on the expression and survival patterns. **E** After the HIF-2α was knocked down, the mRNA level of hsa-mir-7-5p was detected using qRT-PCR. **F** After using the mimics of miR-7-5p, the expression level of miR-7-5p was detected using PCR, and the TBC1D5 protein level was detected using Western Blot. **G** After using the inhibitor of miR-7-5p, the expression level of miR-7-5p was detected using PCR, and the TBC1D5 protein level was detected using Western Blot. **H** ChIP experiment results of potential HIF-2α binding sites in the miR-7-5p promoter are based on the HIF-2α binding sequence. **I** The luciferase assay results. The truncation of the promoter showed that miR-7-5p bound to the TBC1D5 at position 2912–2918 of TBC1D5 3′ UTR. *P < 0.05, **P < 0.01, ***P < 0.001, ****P < 0.0001. *NS* no significance

As shown earlier, a part of our screening datasets contains the sequencing data of HIF-2α knockdown RCC cell line 786-O. After stably knocking down HIF-2α, whole transcriptome sequencing revealed 1778 significantly upregulated genes and 1808 significantly downregulated genes, and the expression of TBC1D5 was significantly elevated (Fig. 6A). The protein expression of TBC1D5 was also upregulated in ccRCC cell lines A498 and 786-O after HIF-2α knockdown (Fig. 6B). Considering that HIF-2α often acts as a transcription factor, and the expression change of TBC1D5 is opposite to that of HIF-2α, we assume a miRNA might connect two genes. We used sequencing data, TargetScan, and Starbase to screen the potential miRNA. As a result, hsa-miR-7-5p and has-miR-495-3p were regulated by HIF-2α and could target TBC1D5 mRNA simultaneously (Fig. 6C). For further selection, we investigated the different expression levels of these two miRNAs in tumor and normal tissue, a correlation of two miRNAs and HIF-2α, and their clinical prognostic significance. Finally, hsa-miR-7-5p was identified as the object for further validation (Fig. 6D). In ccRCC, hsa-miR-7-5p had a relatively lower expression in normal tissue than in tumor tissue, and the expression is significantly correlated with HIF-2α. The expression of miR-7-5p was significantly decreased after HIF-2α knockdown in ccRCC cell lines (Fig. 6E). After transfection mimic of miR-7-5p in both cell lines, protein levels of TBC1D5 significantly reduced. However, protein levels of TBC1D5 were increased after inhibiting miR-7-5p transfection (Fig. 6F, G). After searching the hsa-miR-7-5p promoter region sequence, we found only one potential Hif-2α binding Site, ACGTG. We used ChIP experiments in the A498 cell line to verify the binding Site. The results showed that when containing an ACGTG sequence, HIF-2α could bind to the promoter region. However, for control sequences without ACGTG, there was no significant difference in quantitative polymerase chain reaction (qPCR) results between the ChIP and immunoglobulin (Ig) G groups (Fig. 6H). This showed that the promoter region of miR-7-5p contains the HIF-2α binding site sequence; therefore, HIF-2α could regulate the miR-7-5p expression. We also searched a potential sequence of miR-7-5p binding to TBC1D5 at position 2912–2918 of TBC1D5 3′ UTR, and luciferase experiments in the A498 cell line were performed. The results showed that the fluorescence signal intensity of TBC1D5 in the wild-type (WT) group significantly decreased after transfection with a mimic of miR-7-5p. The transfection of the mimic had no significant effect on the expression of TBC1D5 while in the sequence mutation (MUT) group, indicating the targeted binding of miR-7-5p to TBC1D5 (Fig. 6I). Therefore, we validated a new regulatory mechanism of HIF-2α in ccRCC by regulating has-miR-7-5p, which influences TBC1D5 expression.

### TBC1D5 inhibits the progression of ccRCC in vivo

**Fig. 7:**
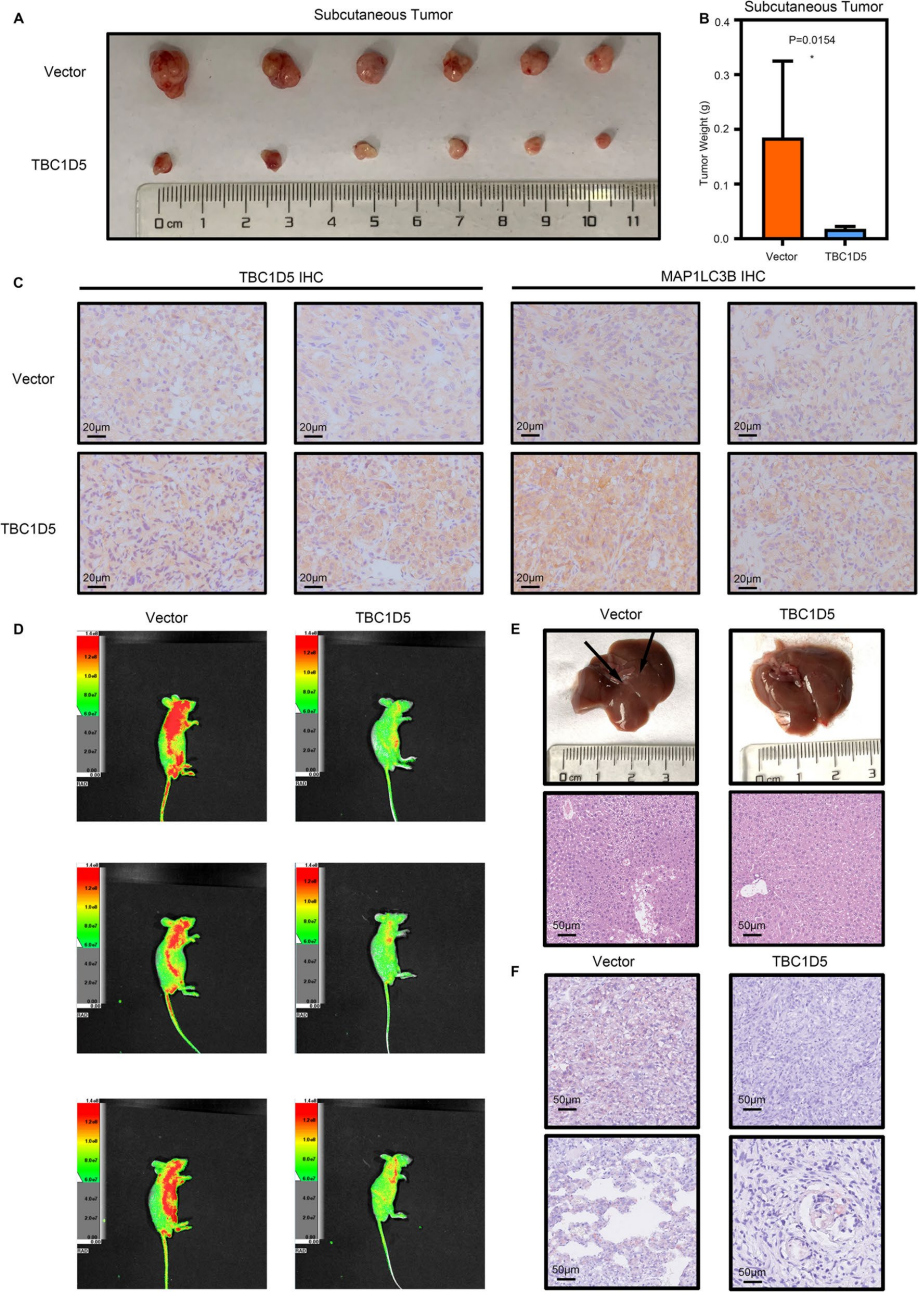
TBC1D5 inhibits the progression of ccRCC in vivo. **A** Subcutaneous implantation mouse models were established using vector or TBC1D5-overexpression cells. **B** Tumor weight of xenografts were measured and compared between vector and TBC1D5-overexpression group after 45 days. **C** IHC staining of TBC1D5 and MAP1LC3B was performed in the tumor sections from mouse models that were established using cells transfected with vector and TBC1D5-overexpression lentivirus. Scale bar: 20 μm. **D** Living fluorescence images of the metastasis model in the TBC1D5 overexpression and control groups. **E** The specimen and hematoxylin and eosin (HE) staining of liver metastasis of tumor. Scale bar: 50 μm. **F** Oil red staining of subcutaneous tumors specimen. Scale bar: 50 μm. *P < 0.05

Based on the in vitro experiment results, we conducted in vivo experiments to further validate the anticancer effect of TBC1D5 in ccRCC. The A498 cell line with stable TBC1D5 overexpressions or control cells was subcutaneously injected into mice to observe the tumor occurrence and progression. After 45 days, the mice were dissected, and the tumors were weighed. We found that the size and weight of subcutaneous xenografts in the TBC1D5 overexpression group were significantly smaller and lower than those in the control group (Fig. 7A, B). Besides, immunohistochemical staining on sections of subcutaneous tumors showed increased expression of TBC1D5 and MAP1LC3B in the TBC1D5 overexpression group.

However, the lipid accumulation was significantly reduced, indicating elevated autophagy levels in TBC1D5 overexpressed tumors (Fig. 7C). Furthermore, we evaluated the tumor metastatic capacity in vivo through a tail vein injection metastasis model in nude mice. In vivo imaging showed that the sizes of metastatic lesions were smaller in the TBC1D5 overexpression group than in the control group (Fig. 7E). After dissecting the livers of the mice, the liver of the control mice showed white spots of metastasis, whereas we found no metastasis in the TBC1D5 overexpression group (Fig. 7E). Oil red staining of subcutaneous tumors showed that lipid accumulation was significantly reduced after TBC1D5 overexpression (Fig. 7F).

**Fig. 8:**
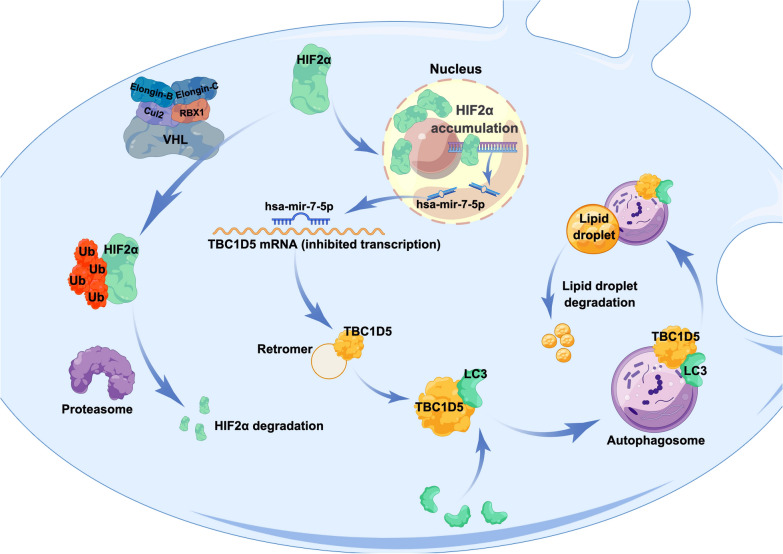
A model of HIF2α affecting autophagy through TBC1D5 in ccRCC

Overall, the specific mechanism was: HIF-2α acts as a transcription factor and promotes the expression of hsa-mir-7-5p, hsa-mir-7-5p bind to 3′ UTR in TBC1D5 mRNA to inhibit TBC1D5 transcription. TBC1D5 suppresses the progression and lipid accumulation of ccRCC by regulating the level of autophagy in tumor cells (Fig. 8).

## Discussion

RCC is the top three most common malignant tumors in the urinary system [1]. However, ccRCC is RCC’s most common histological classification [4]. ccRCC is insensitive to conventional chemotherapy, radiotherapy, and interferon therapy; therefore, advanced ccRCC is mainly treated using specific molecule-targeted drugs or a combination of targeted drugs and immunotherapy [29–32]. However, the limited survival time, high cost, and serious side effects still deeply affect patients. Therefore, it is necessary to further explore ccRCC’s mechanism and provide potential new targets for its treatment.

High expression of HIF-2α and low autophagy level facilitate lipid accumulation and ccRCC prognosis. This suggests a potential link between the HIF-2α pathway and autophagy in ccRCC, confirmed by our preliminary experiment. There is no previous research on this topic; therefore, we tried to find the specific mechanism that connects the HIF-2α pathway and autophagy. Through bioinformatics analysis selection, we found that TBC1D5 was closely correlated with HIF-2α and autophagy. TBC1D5 belongs to the TBC family of proteins. TBC family proteins containing the TRE2/Bub2/CDC16 domain are highly conserved in eukaryotic cells [33]. Commonly, these proteins were considered as GTPase activating proteins (GAPs) of the Rab small GTPase family proteins [34, 35]. TBC1D5 is lowly expressed in ccRCC, and the expression level is significantly associated with prognosis. TBC1D5 overexpression in ccRCC can suppress tumor cells’ proliferation, migration, and invasion abilities.

TBC1D5 mainly acts as GAPs of RAB7 [26]. As a membrane trafficking protein, RAB7 is highly involved in the late endocytic fusion, mitophagy, fusion of the autophagosome to the lysosome, and autophagic degradation of lipid droplets. TBC1D5 and retromer complex can control the activity, localization, and mobility of RAB7, further regulating the biological process of RAB7 involved, especially those correlated with autophagy. Moreover, previous studies certified that TBC1D5 can promote autophagy at an early stage by directly binding to MAP1LC3. The induction of enhanced autophagy fosters the transfer of TBC1D5 from the retromer to the autophagosome membrane, whereas silencing TBC1D5 significantly inhibits autophagosome formation [27]. Furthermore, TBC1D5 can directly act with autophagy-related proteins ULK1 and ATG9, regulating mitophagy initiation through its GAP activity [36]. Our experiments verified that in ccRCC, the facilitating effect of TBC1D5 on autophagy still exists.

The selection procedure of TBC1D5 was derived from sequencing data of the HIF-2α knockdown RCC cell line. Sequencing data showed that HIF-2α knockdown elevated TBC1D5 expression level in ccRCC, and this result was further confirmed by our experiments. Considering that HIF-2α often acts as a transcription factor, and the expression change of TBC1D5 contrasts with that of HIF-2α, we assume two genes might be connected with a miRNA. miRNA is a class of non-coding RNA sequences of approximately 22 bases in size, silencing target gene expressions at the post-transcriptional level. After the screening, we considered hsa-mir-7-5p as the candidate due to its expression pattern and the correlation with clinical patterns.

Furthermore, we found a potential HIF-2α binding site in the promoter region of miR-7-5p and a potential binding site of miR-7-5p and TBC1D5 3′ UTR. ChIP and luciferase experiments proved both binding sites mentioned above, showing a novel pathway of how HIF-2α regulates autophagy. Notably, our rescue experiments demonstrated that TBC1D5 is vital in the tumor-promoting effects of HIF-2α since TBC1D5 knockdown reversed decreased proliferation, migration, invasion, and lipid accumulation caused by HIF-2α knockdown.

The essential role of abnormal lipid metabolism in promoting ccRCC’s proliferation and progression has been studies earlier; autophagic degradation of lipid droplets can significantly reverse this process [37, 38]. No previous research has focused on the connection between autophagic lipid degradation and the most representative gene alteration of HIF-2α in ccRCC. Our study is the first to demonstrate the specific mechanisms of HIF-2α lipid metabolism by regulating autophagy and further influence tumor progression in ccRCC.

In conclusion, HIF-2α promotes hsa-mir-7-5p expression and inhibits TBC1D5’s expression level in ccRCC, leading to reduced autophagy levels and the promotion of lipid droplet deposition, which ultimately promotes ccRCC progression. This study is the first to determine the specific mechanism of HIF-2α in negatively regulating autophagy in ccRCC and discover the connection of HIF-2α/hsa-mir-7-5p/TBC1D5. We hope our findings bring new ideas to the pathogenesis of HIF-2α related ccRCC, thereby providing new targets for diagnosis and treatment.

## Materials and methods

### Sequencing of the whole transcriptome

Oebiotech, China, supported the whole transcriptome sequencing following HIF-2α stable knockdown (contract number = OE2017H0149S). The mirVana miRNA isolation kit (Ambion) was used for RNA extraction. Then, the Agilent 2100 Bioanalyzer (Agilent Technologies, Santa Clara, CA, USA) was used to evaluate RNA integrity and obtain samples with RNA integrity number (RIN) ≥ 7. Next, the gene library was built using the TruSeq Stranded mRNA LTSample Prep Kit (Illumina, San Diego, CA, USA). Finally, the Illumina sequencing platform (HiSeqTM 2500 or Illumina HiSeq X Ten) was used to obtain 125 bp/150 bp paired-end reads for sequencing analysis.

### Human ccRCC tissues and cell lines

This study’s clinical specimens were obtained from the Department of Urology, Wuhan Union Hospital, between 2019 and 2020. Notably, all the specimens were postoperative pathological specimens diagnosed as ccRCC and normal adjacent tissue. Patients had not undergone any targeted therapy or chemoradiotherapy before the operation. Each pair of specimens was divided into two groups. One group was immediately frozen in liquid nitrogen, and the other was fixed in formalin. The former was used for extracting protein and RNA, and the latter for paraffin embedding and immunohistochemical staining. The Huazhong University of Science and Technology approved the collection of tissue specimens, and informed consent was obtained from the patients and their families. Human RCC cell lines 786-O and A498 were obtained from the American Type Culture Collection (ATCC, Manassas, VA, USA). The DMEM high glucose medium containing 10% fetal bovine serum and 1% penicillin–streptomycin was prepared for cell culture. The incubator set in a 5% CO_2_ at 37 °C.

### Immunohistochemistry and HE staining

Tissue samples embedded in formalin-fixed paraffin were cut into 4-µm pieces for immunohistochemical staining. Following paraffin removal with ethylenediaminetetraacetic acid and rehydration, slices were incubated at 120 °C for 5 min for antigen retrieval. Slices were incubated with 3% H_2_O_2_ for 15 min at room temperature (25 °C). Fetal bovine serum was then used to block the slices. After serum removal, slices were incubated in primary antibody solution at 4 °C overnight, followed by DAKO secondary antibody solution at room temperature for 1 h. Subsequently, slices were added to freshly prepared DAB reagent and observed under a microscope. Finally, slices were counterstained in hematoxylin. Immunohistochemical staining uses the same primary antibody as that used in western blot. Immunohistochemical staining of subcutaneous tumors in mice was performed at 1:450 dilution of TBC1D5 and 1:200 dilution of LC3B and at 1:200 dilution of TBC1D5 and 1:200 dilution of human RCC.

For hematoxylin and eosin (HE) staining, paraffin was removed with xylene and treated with anhydrous ethanol and 95%, 85%, and 75% ethanol, respectively. Then, slices were stained with hematoxylin, treated with 1% hydrochloric acid, and stained with eosin. After drying, slices were treated with xylene and sealed.

### Virus transfection

The cells were cultured in a six-well plate with a confluency of approximately 40%, and the medium was changed into a serum-free medium. Notably, 40 µL transfection reagent A (Genechem, China), transfection reagent P (Genechem, China), and 40 Moi lentivirus were added to transfect the virus. After 8 h, the cells were cultured in a serum-containing medium. After 3 days, puromycin was added for screening. After 3 days of continuous puromycin treatment, the protein was extracted to verify the transfection efficiency.

### Transwell experiment

Cells were cultured in a serum-free medium for 24 h to test the migratory and invasion ability. Then, cells were collected and suspended in a serum-free high-glucose medium. For the invasion assay, Matrigel and high-glucose medium were diluted at a ratio of 1:8 and placed on the upper Transwell inserts at 37 °C for 2 h. Furthermore, 6 × 10^4^ A498 cells and 8 × 10^4^ 786-O cells were added into the upper chamber. For the migration assay, 3 × 10^4^ A498 cells and 4 × 10^4^ 786-O cells were added into the upper chamber without Matrigel. Serum-containing cell culture medium was added into the lower chamber and incubated for 24–48 h in both assays. Next, cells were fixed in anhydrous methanol and stained with crystal violet. The cells were observed under a microscope, and multiple fields of view were collected randomly. The cells were counted and analyzed statistically.

### Cell counting kit-8 assays

The cells used in the experiment were collected and counted. Notably, 96-well plates were utilized to measure the number of alive cells, and 2 × 10^3^ cells were seeded in each well. After incubating for 0, 24, 48, 72, and 96 h, the CCK8 assay (YEASEN, China) was used to detect the number of alive cells. Then, all the data were counted and analyzed statistically.

### RNA isolation and real-time PCR quantitative analysis

TRizol reagents (Thermo Fisher Scientific, Waltham, MA, USA) were used to extract total RNA from tissue and cells. After chloroform, isopropanol, and ethanol treatment, samples were dissolved in Diethyl pyrocarbonate (DEPC) water. A 2-µL solution was used to confirm the concentration and purity of extracted RNA using a NanoDrop2000 spectrophotometer (NanoDrop Technologies, USA). Accordingly, RNA was diluted to 250 ng/µL at − 80 °C for storage.

We used 4 µL above the RNA sample for reverse transcription, followed by qPCR analysis through the SYBR Green mix (YEASEN, China) in StepOnePlus Real-Time polymerase chain reaction (PCR) System (Thermo Fisher Scientific). Glyceraldehyde 3-phosphate dehydrogenase (GAPDH) is used as an internal reference in PCRs associated with proteins, and U6 is used in PCRs associated with microRNAs.

### Protein extraction and Western blot

The radioimmunoprecipitation assay lysate (Wuhan Servicebio technology), Protease inhibitor Cocktail, and phenylmethylsulfonyl fluoride were prepared in a 98:1:1 ratio for protein extraction from both tissue and cells. The protein concentration of extracted samples was measured with a bicinchoninic acid quantitative reagent (Beyotime Institute of Biotechnology, P0012S, China).

Notably, 40 µg of the above-extracted protein were subjected to sodium dodecyl sulfate–polyacrylamide gel electrophoresis for gel electrophoresis, which was subsequently transferred to a polyvinylidene fluoride (PVDF) membrane by Roche (03010040001, Basel, Switzerland). After that, 5% skimmed milk was used to block the PVDF membrane for 1 h at room temperature. Then, the membrane was incubated with the primary antibodies overnight at 4 °C. After rinsing with phosphate-buffered saline with Tween 20 thrice, the membrane was incubated with the secondary antibodies overnight at room temperature. A ratio of 1:1000 was used to dilute the primary antibody, and 1:2000 was used to dilute the secondary antibody. The primary antibodies used were TBC1D5 (Proteintech, 17078-1-AP), HIF-2α (Abclonal, A7553), GAPDH (Abclonal, A19056), and LC3B (Abclonal, A19665). The secondary antibodies used were horseradish peroxidase (HRP)-conjugated Affinipure Goat Anti-Rabbit IgG (H+L) (Proteintech, SA00001-2) and HRP-conjugated Affinipure Goat Anti-Mouse IgG (H+L) (Proteintech, SA00001-1).

### Oil red staining

Oil Red was mixed with ultrapure water at a ratio of 3:2 in advance, and the supernatant was collected for staining. Cell confluence in Oil red staining is approximately 30%. After removing the cell culture medium, cells were rinsed with PBS thrice and fixed with 4% paraformaldehyde. Then, cells were stained with prepared oil red dye for 30 min at room temperature. Finally, rinse it with PBS, dry it off, and observe under a microscope (Olympus, Japan, Tokyo).

### Chromatin immunoprecipitation (ChIP) assay

ChIP assay was performed using the CST-SimpleChIP Enzymatic Chromatin IP Kit (Agarose Beads). RiboBio (RiboBio, Guangzhou, China) was constructed using the plasmids with mutated TBC1D5 promoter region. Pretreatment of the cell lysate and Chip-Grade protein G Agarose Beads (CST, 9007S, Boston, MA, USA) was conducted by Rabbit IgG (CST, 2729, Boston, MA, USA). An anti-HIF-2α (Abclonal, A7553) was incubated overnight at 4 °C. IgG was used as a negative control.

The relevant target sequence of primers designed for miR-7-5p promoter used for CHIP analysis is shown as follows:

### Mimic/inhibitor

The sequence information of miRNA mimics and inhibitors comes from miRbase (www.miRbase.org) and is constructed using RiboBio (RiboBio, Guangzhou, China). A498 and 786-O cells were transfected with mimic/inhibitor and control reagent. Notably, some cells were collected after 24 h of incubation, and RNA was extracted to verify the transfection efficiency using reverse transcription (RT)-qPCR. After 48 h, the remaining cells were collected, and the protein was extracted to verify the effect of miR-7-5p on TBC1D5 using a western blot.

### Luciferase experiment

TBC1D5-related double luciferase plasmid was constructed using RiboBio (RiboBio, Guangzhou, China) based on the vector pmiR-RB-Report™. A498 cells were cultured in a 48-well plate, and Lipofectamine 2000 was used to transfect luciferase plasmid and mimic/control reagent into A498 cells. After 24 h, the cells were collected, and the fluorescence intensity was detected with the Dual-Luciferase Assay reagent (Promega, E1910). The ratio of the fluorescence intensity of the firefly to the intensity of Renilla was used as a parameter to reflect the reporter gene’s activity.

### Tumor formation assay and in vivo cancer metastasis assay

Nude mice were divided into the control group and the TBC1D5 overexpression (TBC1D5) group, with nine mice in each group. In each group, six nude mice were injected with tumor cells subcutaneously for tumor formation assay, and the remaining three were injected with tumor cells through the caudal vein to establish metastatic tumor models. The amount of cells injected subcutaneously was 2 × 10^6^, and the number of cells for caudal vein injection was 1 × 10^6^. The general condition and subcutaneous tumor size were observed every 3 days. After 45 days, 12 mice in the subcutaneous injection group were dissected, and the weight and size of the subcutaneous tumors were measured; six mice with metastatic tumors were dissected, and the liver was fixed.

### Tumor xenografts

Notably, 4–5-week-old male BALB/c nude mice were selected for the tumor xenograft experiments. Furthermore, 786-O cells, labeled with Cy3 and transfected with overexpressed TBC1D5 plasmids or control vectors, were subcutaneously injected into the tail veins of the mice (3 × 10^6^, 200 μL), who were sacrificed after 6–7 weeks. No blinding occurred during the animal experiments. Xenograft images of the mice were obtained using the In-Vivo FX PRO (BRUKER Corporation, USA). The Animal Research Committee of the Academic Medical Centre at Huazhong University of Science and Technology reviewed and approved this study's animal experiments. Care and handling of the animals followed the guidelines of Institutional and Animal Care and Use Committees.

### Transmission electron microscopy

Fresh tissue samples were fixed in 2.5% phosphate‐buffered glutaraldehyde and 1% phosphate‐buffered osmium tetroxide. The samples were embedded, sectioned, double‐stained with uranyl acetate and lead citrate, and visualized using an H‐7650 transmission electron microscope (Hitachi, Japan).

### Bioinformatics analysis

The KEGG pathway map (map04140) listed 36 autophagy-related genes; we verified the expression level of the genes using the Kidney Renal Clear Cell Carcinoma (KIRC) data set in the Cancer Genome Atlas (TCGA) database. Based on the KIRC data set, we screened genes expressed differently between tumor and normal tissue (P < 0.05) and significantly expressed, correlating with HIF-2α. These screened genes were used to perform GO and KEGG analysis to determine the potential relationship between HIF-2α and autophagy pathways. Our whole transcriptome sequencing included data from the HIF-2α knockout/parental 786-O cell line and normal/tumor tissue from a ccRCC patient; differentially expressed genes (DEGs) were screened by fold change > 2 and p < 0.05. In addition, three sequencing data from GEO data sets of ccRCC (GSE66270, GSE66271, GSE53757) were analyzed, and DEGs were selected. The intersection of DEGs from our 786-O cell line sequencing data, our tissue sequencing data, the TCGA database, and three GEO data sets was taken. Then, selected genes were further screened based on the annotation of the GO term to determine autophagy-related genes. After all these procedures, DEGs that simultaneously correlated with HIF-2α and autophagy could be obtained. Using the clinical parameters in the TCGA database, Kaplan–Meier curves of OS/DFS and ROC curves were made to help the final selection, and TBC1D5 was selected as our target gene. Then, we searched the KIRC data set in the TCGA database and verified the expression of TBC1D5 in RCC by combining the clinical parameters such as age, TNM, tumor, histologic stages, and OS/DFS state. We ranked TCGA-KIRC samples based on the TBC1D5 expression level, ranging from high to low, with the first 266 samples defined as TBC1D5 high expression and the remaining 265 defined as low expression, the relationship between TBC1D5 expression, prognosis, and other clinical parameters was analyzed. In addition, we searched the GEO database and used the sequencing data of GSE11151 and GSE6344 to verify the expression pattern of TBC1D5 in ccRCC. Gene enrichment analysis was performed using GSEA software v4.1.0 for Windows (UC San Diego, San Diego, USA) to analyze TBC1D5’s function in ccRCC.

### Statistical analysis

All data were analyzed using IBM SPSS Statistics version 22.0 (IBM Corp., Armonk, N.Y., USA) software and presented as the mean ± standard deviation. Differences between groups were evaluated using t-tests. The two groups were compared using a two-tailed Student’s t-test. A one-way analysis of variance was performed to assess the group differences. Statistical significance was set at P < 0.05.

### Supplementary Information

**Additional file 1: Figure S1.** HIF-2α knocks down leading to increased LC3 level. A. The protein level of LC3 was detected using Western Blot after knocking down HIF-2α.

**Additional file 2: Figure S2.** Kaplan–Meier curves of overall survival (OS) for five candidate genes. A. Kaplan–Meier curves of OS based on TBC1D5 expression in patients with ccRCC. B. Kaplan–Meier curves of OS based on IFT88 expression in patients with ccRCC. C. Kaplan–Meier curves of OS based on ATM expression in patients with ccRCC. D. Kaplan–Meier curves of OS based on PDGFA expression in patients with ccRCC. E. Kaplan–Meier curves of OS based on HK2 expression in patients with ccRCC.

**Additional file 3: Figure S3.** Kaplan–Meier curves of DFS and OS based on TBC1D5 expression in patients with different malignancy grade ccRCC. A. Kaplan–Meier curves of DFS according to TBC1D5 expression in T1 or T2 patients with ccRCC. B. Kaplan–Meier curves of DFS according to TBC1D5 expression in N1 and Nx patients with ccRCC. C. Kaplan–Meier curves of DFS according to TBC1D5 expression in patients with stage 1 and 2 ccRCC. D. Kaplan–Meier curves of DFS according to TBC1D5 expression in patients with histo-grade 3 and 4 ccRCC. E. Kaplan–Meier curves of OS according to TBC1D5 expression in patients with M0 ccRCC. F. Kaplan–Meier curves of OS according to TBC1D5 expression in patients with N1 and Nx ccRCC. G. Kaplan–Meier curves of OS according to TBC1D5 expression in patients with N0 ccRCC. H. Kaplan–Meier curves of OS according to TBC1D5 expression in patients with histo-grade 3 and 4 ccRCC. *P < 0.05, **P < 0.01, ***P < 0.001.

**Additional file 4: Figure S4.** The expression level of TBC1D5 is comparable in adjacent tissue of different malignancy grade ccRCC. A. The mRNA levels of TBC1D5 shows no difference in adjacent tissue between N0 and N1 or Nx patients. B. The mRNA levels of TBC1D5 shows no difference in adjacent tissue between living and deceased patients. C. The mRNA levels of TBC1D5 shows no difference in adjacent tissue between disease-free and progressed patients. D. mRNA levels of TBC1D5 shows no difference in adjacent tissue between different T stage patients. E. mRNA levels of TBC1D5 shows no difference in adjacent tissue between different histologic grade patients. There are only 2 patients in T4 stage and 1 patient in histologic grade 1, so we put together T3 and T4 stage, as well as grade 1 and 2 for better statistics.

**Additional file 5: Figure S5.** Semi-quantification of oil-red staining. A. FIG3.G: lipid droplet accumulation in ccRCC cells was significantly decreased by TBC1D5 overexpression. B. FIG4.G: chloroquine promote lipid accumulation in ccRCC, rapamycin inhibit lipid accumulation in ccRCC, chloroquine reverse the inhibitory effect by TBC1D5 overexpression, while rapamycin is in synergy with TBC1D5 overexpression. C. FIG5.E: lipid accumulation in cells significantly reduced after HIF-2α knockdown, knockdown of TBC1D5 significantly increased lipid accumulation, knockdown of both HIF-2α and TBC1D5 can reverse the decreased lipid accumulation caused by knockdown of HIF-2α alone. *P < 0.05, **P < 0.01, ***P < 0.001.

## Data Availability

The raw data supporting the conclusions of this article will be made available by the authors, without undue reservation.
