# Correction to: TBC1D5 reverses the capability of HIF‑2 in tumor progression and lipid metabolism in clear cell renal cell carcinoma by regulating the autophagy

**DOI:** 10.1186/s12967-024-05189-5

**Published:** 2024-05-27

**Authors:** Yu Huang, Zhiyong Xiong, Jianjun Wang, Yafen Gao, Qi Cao, Decai Wang, Jian Shi, Zhixian Chen, Xiong Yang

**Affiliations:** 1 Department of Urology, Tongji Medical College, Union Hospital, Huazhong University of Science and Technology, Wuhan, China; 2 Department of Hepatobiliary Surgery, School of Medicine, Mianyang Central Hospital, University of Electronic Science and Technology of China, Mianyang, China; 3 Department of Anesthesiology, Union Hospital, Tongji Medical College, Huazhong University of Science and Technology, Wuhan, China; 4 Department of Urology, School of Medicine, Mianyang Central Hospital, University of Electronic Science and Technology of China, Mianyang, China; 5 Departments of Pathology, Li Ka Shing Faculty of Medicine, School of Clinical Medicine, The University of Hong Kong, Pok Fu Lam, Hong Kong China

**Journal of Translational Medicine (2024) 22:212**

10.1186/s12967-024-05015-y

Following publication of the original article [1], we have been notified that the authors below were not marked as corresponding authors.

It is now:

Yu Huang1†, Zhiyong Xiong1†, Jianjun Wang2†, Yafen Gao3, Qi Cao1, Decai Wang4, Jian Shi1, Zhixian Chen5 and Xiong Yang1*

It should be as per below:

Yu Huang1†, Zhiyong Xiong1†, Jianjun Wang2†, Yafen Gao3, Qi Cao1, Decai Wang4, Jian Shi1*, Zhixian Chen5* and Xiong Yang1*

The original article was updated.
