# Supplementary figures and images for: TBC1D5 reverses the capability of HIF-2α in tumor progression and lipid metabolism in clear cell renal cell carcinoma by regulating the autophagy
Source: J Transl Med. 2024 Feb 28;22:212. doi: 10.1186/s12967-024-05015-y (PMC10900628; doi:10.1186/s12967-024-05015-y)

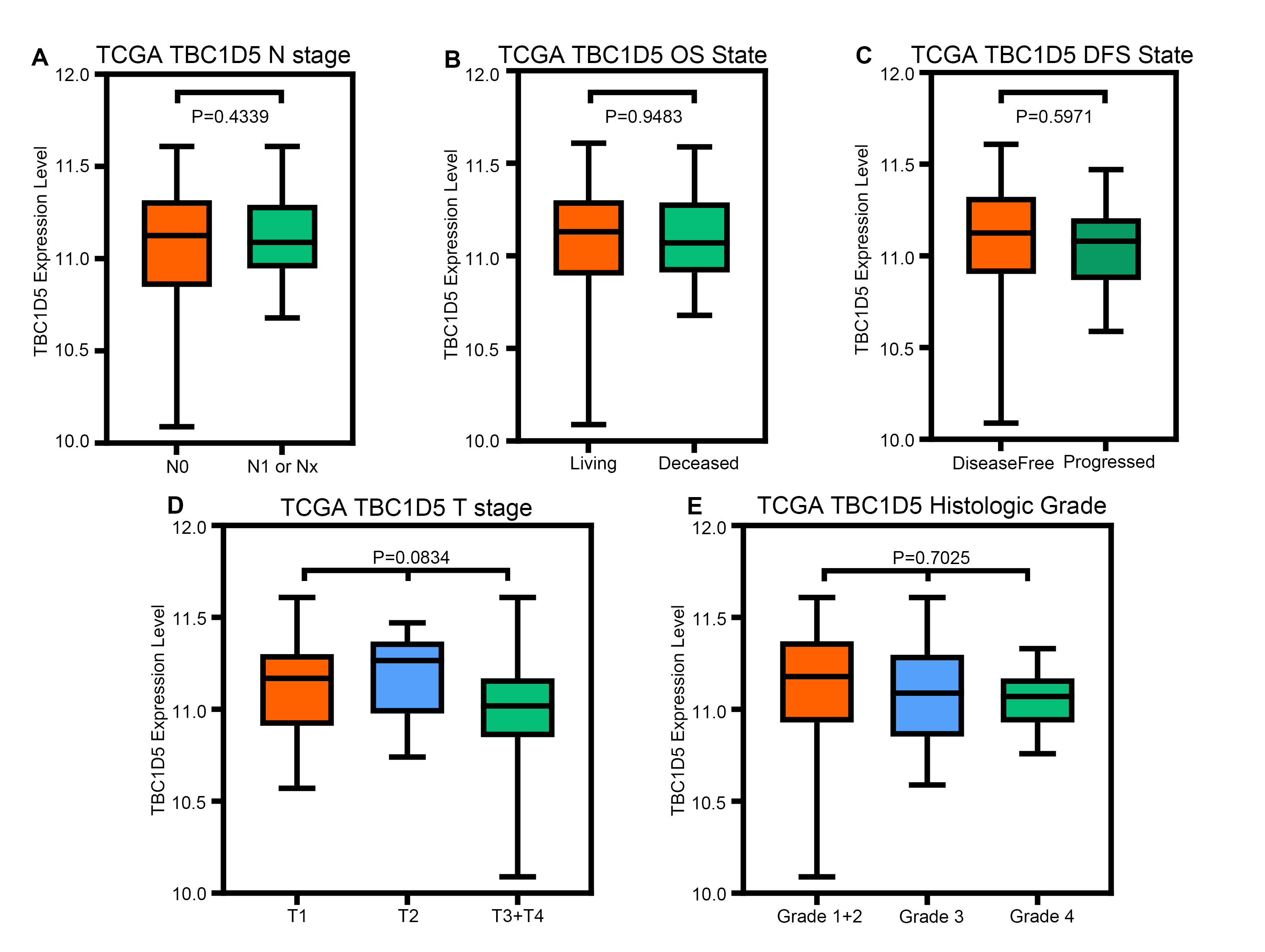

Supplement: Supplementary file 4 — Additional file 4: Figure S4. The expression level of TBC1D5 is comparable in adjacent tissue of different malignancy grade ccRCC. A. The mRNA levels of TBC1D5 shows no difference in adjacent tissue between N0 and N1 or Nx patients. B. The mRNA levels of TBC1D5 shows no difference in adjacent tissue between living and deceased patients. C. The mRNA levels of TBC1D5 shows no difference in adjacent tissue between disease-free and progressed patients. D. mRNA levels of TBC1D5 shows no difference in adjacent tissue between different T stage patients. E. mRNA levels of TBC1D5 shows no difference in adjacent tissue between different histologic grade patients. There are only 2 patients in T4 stage and 1 patient in histologic grade 1, so we put together T3 and T4 stage, as well as grade 1 and 2 for better statistics. [file 12967_2024_5015_MOESM4_ESM.tif]

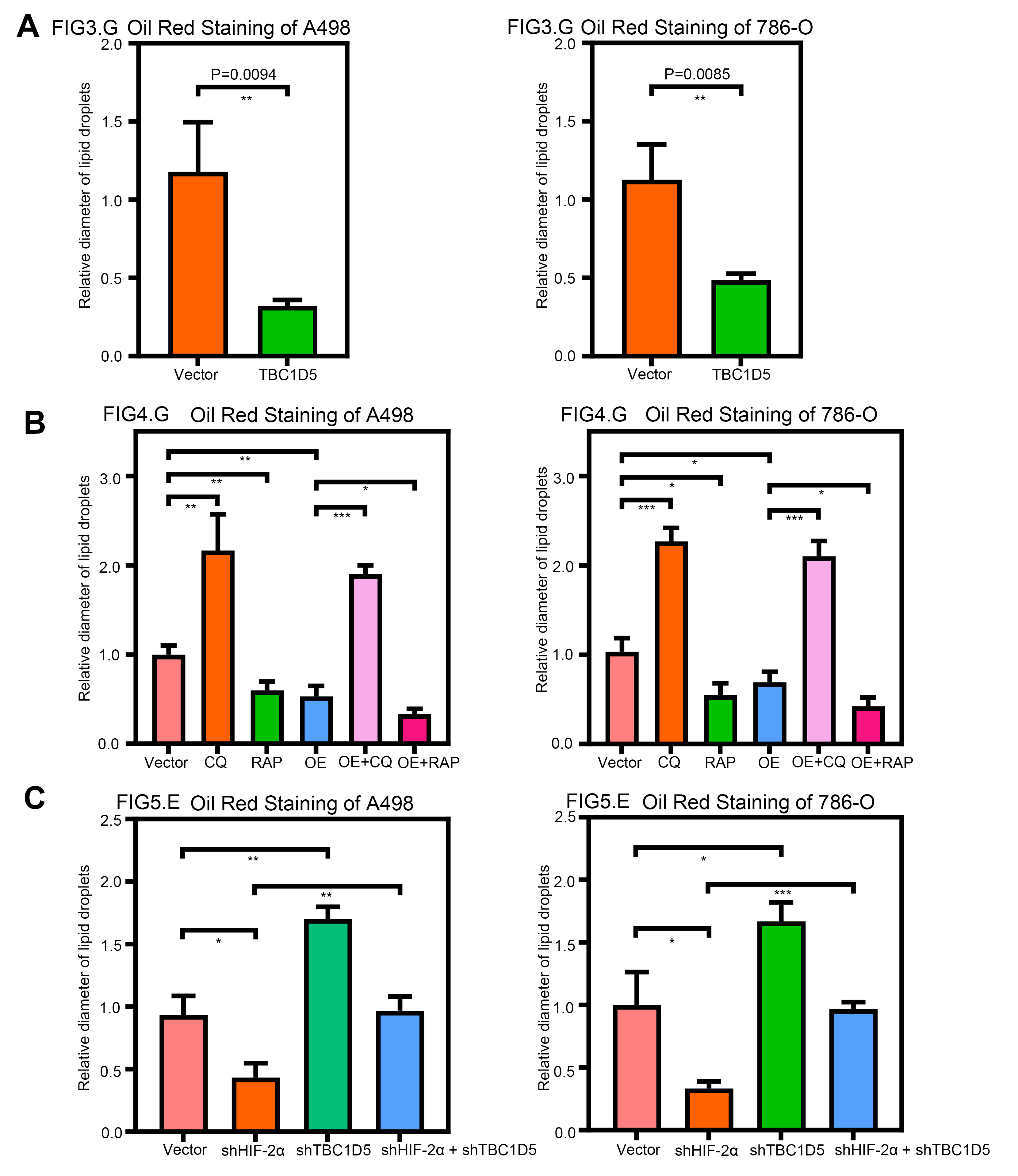

Supplement: Supplementary file 5 — Additional file 5: Figure S5. Semi-quantification of oil-red staining. A. FIG3.G: lipid droplet accumulation in ccRCC cells was significantly decreased by TBC1D5 overexpression. B. FIG4.G: chloroquine promote lipid accumulation in ccRCC, rapamycin inhibit lipid accumulation in ccRCC, chloroquine reverse the inhibitory effect by TBC1D5 overexpression, while rapamycin is in synergy with TBC1D5 overexpression. C. FIG5.E: lipid accumulation in cells significantly reduced after HIF-2α knockdown, knockdown of TBC1D5 significantly increased lipid accumulation, knockdown of both HIF-2α and TBC1D5 can reverse the decreased lipid accumulation caused by knockdown of HIF-2α alone. *P < 0.05, **P < 0.01, ***P < 0.001. [file 12967_2024_5015_MOESM5_ESM.tif]
